# Sellar masses: diagnosis and treatment

**DOI:** 10.3389/fopht.2022.970580

**Published:** 2022-11-24

**Authors:** Dana Al-Bader, Alya Hasan, Raed Behbehani

**Affiliations:** ^1^ Al-Bahar Ophthalmology Center, Ibn Sina Hospital, Ministry of Health, Kuwait City, Kuwait; ^2^ Department of Neurosurgery, Ibn Sina Hospital and Jaber Al-Ahmed Hospital, Ministry of Health, Kuwait City, Kuwait

**Keywords:** pituary adenoma, craniopharingioma, meningioma, optical coherance tomography, compressive optic neuropathy, ganglion cell complex (GCC), sellar masses

## Abstract

Sellar mases can cause a variety of neuro-ophthalmic manifestations, including compressive optic neuropathy, chiasmal syndrome, and ophthalmoplegia due to cranial nerve palsy. Diagnosis involves a thorough history, neuro-ophthalmic examination, and ancillary tests and investigations. Visual field testing is critical in diagnosing and localizing the lesion and determining the extent of visual field loss. Appropriate neuro-imaging is essential in characterizing and localizing the lesion. Neuro-ophthalmologic assessment include meticulous clinical examination and ancillary tests including,visual field testing, which is useful in localizing the lesion, and optical coherence tomography, which is helpful in assessing the degree of axonal and neuronal loss and predicting the visual outcome. Treatment requires a multidisciplinary approach by different specialties, including radiologists, neuro-ophthalmologists, and neurosurgeons. The two primary treatment modalities for these tumors are surgery and radiation therapy. We review the main types of sellar lesions, their neuro-ophthalmologic evaluation, and treatment options.

## Introduction

Pituitary adenomas, craniopharyngiomas, aneurysms, astrocytomas, and meningiomas are among the most common lesions in the sellar area, accounting for around 80% of cases.

Magnetic resonance imaging (MRI) and computed tomography with fat-saturated views and (CT) imaging are essential in the localization and characterization of these lesions (1).

Sellar masses can be classified based on anatomical location as intrahypophysial and extrahypophysial lesions. The proximity of these masses to the anterior visual pathways may cause visual loss due to compression. Visual field abnormalities, vision loss, and diplopia are some of the neuro-ophthalmic deficits caused by chiasmal tumors. The primary treatment modalities include surgery with varying approaches and radiotherapy (see **Table 1**).

**Table 1:** Sellar masses, MRI findings, and treatment.

Sellar tumor:	MRI findings:	Treatment:
Pituitary adenoma	Isointense on T1 nd T2 heterogeneous enhancement	Surgery Radiation
Craniopharyngioma	Solid/cystic Note:calcifications need CT to detect	Surgery: Cystic aspiration +/-Radiation
Meningioma	Dural tail sign	Observation depending on the size, growth, and age Surgery +/- Radiation
Astrocytoma/Glioma	Large cystic lesion with brightly enhancing mural nodule or heterogenous enhancement based on WHO grade	Surgery +/-Radiation
Miscellaneous		
Aneurysm	In areas of slower turbulent flow: Flow void and heterogeneous increased signal intensity	Surgical clipping vs. Endovascular coiling
Germ Cell tumors	Soft tissue mass, ovoid or lobulated heterogenous enhancement DWI restricting mass	Radiation Chemotherapy
Hypothalamic Glioma	T1 enlargement iso to hypointense compared to contralateral side T2 hyperintense	Chemotherapy Surgery Radiation
Rathke’s Cleft Cyst	Intracystic micronodule, best seen on T2-weighted images	Observation Surgery
Hamartoma	Soft tissue iso-attenuating to grey matter, absence of calcification or contrast enhancement	Precocious puberty – medical Epilepsy - surgery
Chordoma	Lytic lesion of the clivus, with intra-tumoral septa	Surgery Radiation
Lymphoma	Isointense on T1 and T2 contrast enhancement	Chemotherapy Radiation

## Neuro-ophthalmologic evaluation

### Clinical examination

The neuro-ophthalmic evaluation will assess the afferent and efferent visual systems. Sellar tumors can produce a variety of afferent visual manifestations (optic neuropathy, chiasmal syndrome, and posterior visual pathway lesions) and efferent (ophthalmoplegia due to cranial nerve palsy). The afferent system is assessed by testing the visual acuity, color vision testing using the standard Ishihara color plates, pupillary testing to check for relative afferent pupillary defect and fundus examination, visual field testing, and OCT. The efferent system is assessed by testing the patient’s ocular eye movements (both ductions and versions), cover-uncover testing to detect any tropia from paralytic strabismus from cranial nerve palsy (third, fourth, or sixth cranial nerve), and measuring the orthoptic alignment with prisms and any detect any nystagmus. See-saw pendular nystagmus is classically associated with tumors of the sellar regions, in which one eye elevates and intorts, and the contra-lateral eye depresses and extorts.

### Visual field testing

The pattern of the visual field can help localize the lesion. A lesion at or posterior to the chiasm produces a hemianopic deficit that respects the vertical midline. Junctional scotoma is an ipsilateral central field defect and contralateral superotemporal field defect due to a compressive lesion at the optic nerve and chiasm junction.

Ogra et al., 2014 reported that in patients referred for neurosurgery with pituitary adenoma, the most common defect pattern was bitemporal defects (n=22, 41%), followed by homonymous defects (n=7, 13%). Unilateral visual field deficits were found in 33% of patients with visual field loss, Patients with bitemporal hemianopia had a mean visual acuity of 6/7.5, with half of them having 6/6 vision in both eyes. While bitemporal visual field loss is the most prevalent, unilateral and altitudinal impairments were found in a considerable number of patients (2).

### Optical coherence tomography

Optical coherence tomography (OCT) is vital in assessing axonal and retinal ganglion cell loss by quantitative analysis of the retinal fiber layer and retinal cell ganglion cell complex layer a. Pre-operative OCT can be useful in predicting the visual outcome after surgical decompression of the optic chiasm, which is helpful in counseling patients about their expected outcome following surgical intervention (3). Patterns of ganglion-cell complex (GCC) loss can aid in the diagnosis of anterior visual pathway compressive lesions, particularly chiasmal compression. GCC thinning on OCT can be a useful tool for detecting visual pathway impairment early on, before the retinal nerve fiber layer (RNFL) thinning, and in certain instances, before visual field loss. Visual field testing is psychophysical test and subject to patient’s ability to perform it well. In addition, it is less sensitive than OCT in detecting axonal loss in early compressive lesions of the visual pathways. OCT, however, is a quicker and more objective test and standardized and reproducible test and is more sensitive tool in of detecting compression of the anterior visual pathways. Both tests, however, are useful and complementary to each other in the evaluation of sellar masses compressing the anterior visual pathways.

Moreover, recent studies show that the visual field loss associated with compressive optic neuropathies is correlated with thinning of the ganglion cell layer complex (GCC) (4).

**Figure 1:**
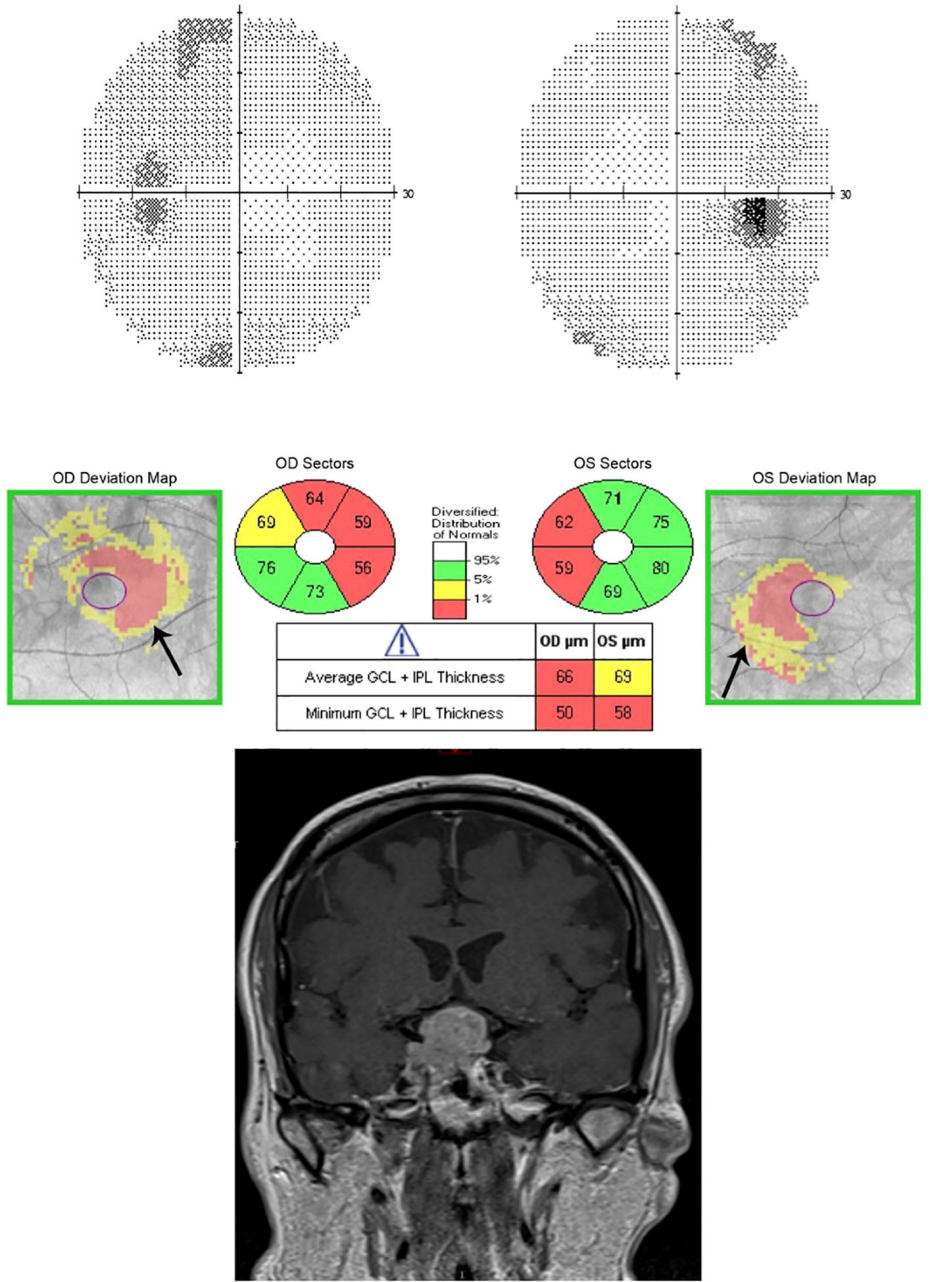
The sensitivity of OCT to detect GCC loss even before visual fields became abnormal for a patient with pituitary adenoma. (Top) Humphrey visual field 24-2 without changes detected in this patient with pituitary macro adenoma. (Middle) Ganglion Cell Analysis of Spectral-domain OCT (Cirrus) showing binasal thinning in the GCC layer despite normal visual fields. (Bottom) MRI T1 coronal view with contrast showing pituitary macro adenoma.

**Figure 2:**
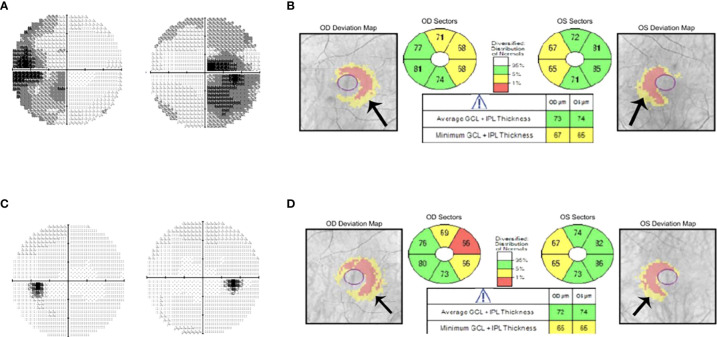
Humphrey visual field 24-2 and OCT with ganglion cell analysis pre- and post-pituitary tumor surgery. **(A)** Humphrey visual field 30-2 showing bitemporal hemianopia in a patient with a pituitary tumor and chiasmal compression. **(B)** Ganglion Cell Analysis of Spectral domain-OCT (Cirrus) shows binasal thinning of the ganglion cell layer with relative overall preservation, indicating a good prognosis for visual recovery following surgical excision of a pituitary tumor. **(C)** Follow-up visual field at one month after surgical decompression of the tumor showing complete recovery of the visual field defect. **(D)** Ganglion cell analysis at 1 month showing persistence of the binasal ganglion cell loss and the overall preservation of the ganglion cell layer thickness.

Pituitary tumors usually affect the crossing nasal retinal fibers and therefore result in preferential ganglion cell loss in the nasal hemiretina respecting the vertical midline even with minimal or no detectable visual field loss (**Figures 1**, **2**) (5).

**Figure 3:**
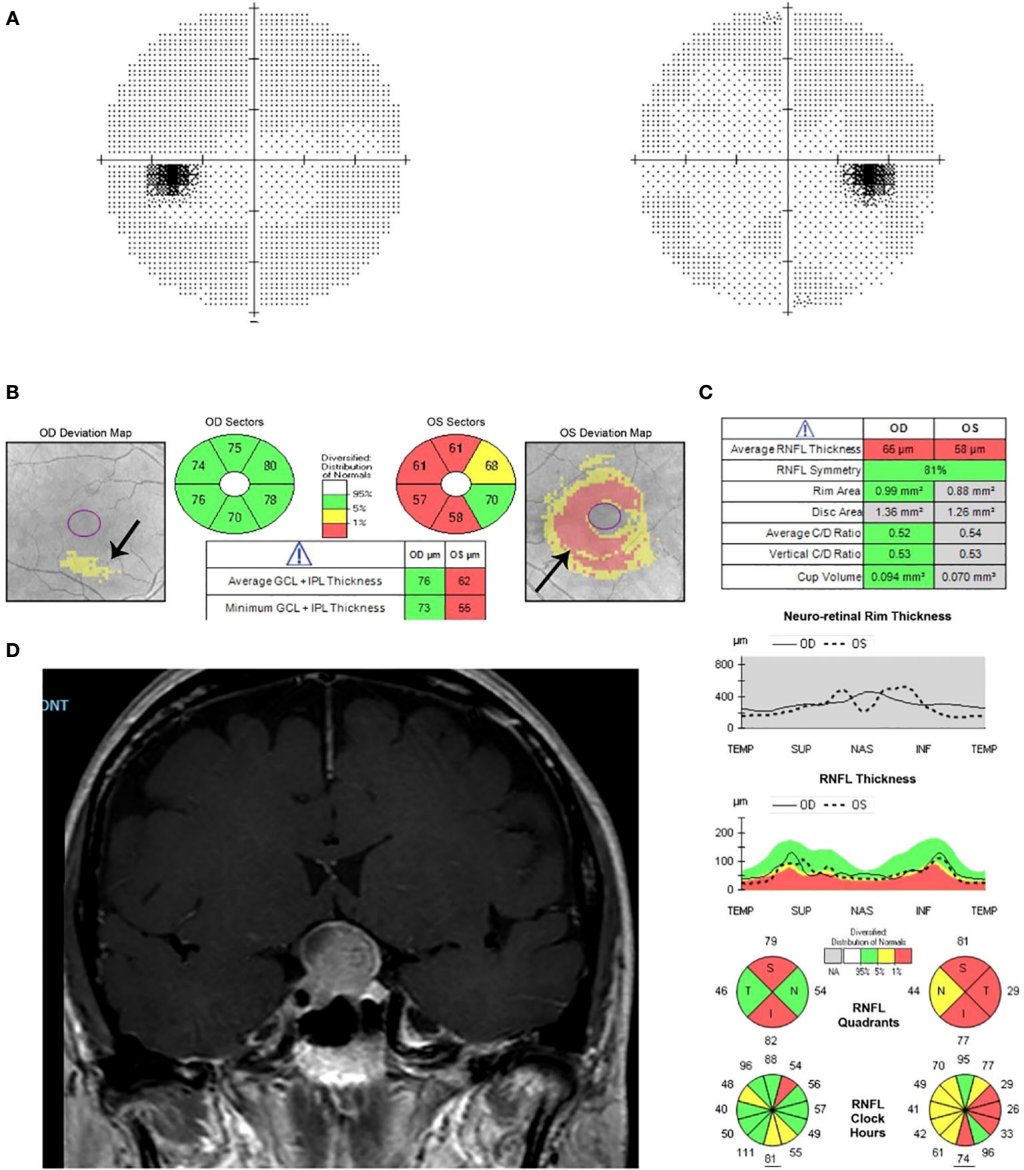
Humphrey visual field 24-2 and OCT with ganglion cell analysis and retinal nerve fiber layer thickness of a patient with sellar and suprasellar meningioma. **(A)** Normal visual field test. **(B)** Evidence of ganglion cell layer loss in the left eye, especially nasally. **(C)** Thinning of the retinal nerve fiber layer in both eyes. **(D)** MRI shows meningioma of the sellar and suprasellar region with homogenous enhancement on T1 with contrast.

Retinal nerve fiber layer thinning is also useful for detecting axonal loss in compressive lesions of the visual pathway (**Figure 3**). The temporal retinal nerve fiber layer quadrant had the strongest positive correlation with the degree of compression and supra-sellar extension in pituitary adenomas (6).

Glebauskiene B et al. reported that when pituitary adenoma patients with suprasellar extension were compared to individuals without suprasellar extension, RNFL thickness was significantly reduced only in the temporal quadrant (p =.009) and had the strongest correlation. Similarly, the proximity between the optic chiasm and the pituitary adenoma was most strongly correlated with the temporal RNFL thickness (r = 0.401, p<.001) (7).

In a multicenter study of 239 patients who had undergone decompression surgery for pituitary tumors, Wang at al found that the inferior RNFL quadrant thickness was the most robust predictor for visual field recovery than the average RNFLT with each 10 μm increase in inferior RNFLT being associated with 1.26 odds ratio for long-term improved visual field recovery (8).

Meyer J et al. assessed the relation between preoperative OCT measures and visual outcomes after neurosurgical decompression of pituitary or parasellar tumors. The study analyzed 108 participants with a mean age of 51.6. It was reported that macular ganglion cell layer (mGCL) was better in predicting long-term visual field recovery, and the superior inner mGCL was the most specific (9).

Yoo et al. have evaluated 79 patients with sellar tumors and have found the the central macular ganglion cell-inner plexiform layer in the inferonasal and superonasal quadrant had a higher predictive and prognostic values for determining post-operative visual recover than the average RNFLT and the papillomacular bundle thickness. Patients with central inferonasal quadrant mGCL thickness greater than 29.75 μm had significant visual field improvement even in the presence of severe preoperative VF loss (10).Tiger et al. studies 23 patients with sellar tumors and compared them to controls and assesses the correlation the preoperative GCC thickness with the post-operative mean deviation of visual fields. It was found that patients with relatively preserved GCC had better visual field improvement with an effect size better than RNFLT (5).

There are several hypotheses for visual recovery following decompression surgery such as axonal injury include direct disruption of conduction through the axon, impairment of anterograde and retro- grade axoplasmic flow, and demyelination with impaired signal con- duction. Immediate recovery of visual function after chiasmal decompression is possibly attributed to the alleviation of a conduction block caused by direct compression, while delayed recovery (weeks to months) is probably a result of restoration of axoplasmic transport or remyelination (11).

## Pituitary adenoma

Pituitary adenomas arise from the anterior pituitary gland (adenohypophysis) can be seen in all age groups. These neoplasms can make up around 14% of intracranial and primary CNS tumors. Pituitary adenomas are growing in incidence (between 3.9 and 7.4 cases per 100,000 per year) and prevalence (76 to 116 cases per 100,000 population) in the general population (about 1 case per 1000 general population), possibly as a result of improved detection with MRI. Thus these are the most common sellar tumors. The most common adenomas diagnosed are prolactin-secreting pituitary adenomas (12). Pituitary tumors may cause a pressure effect on neighboring structures, resulting in hypopituitarism and visual field impairment, which often results in workup leading to their detection. However, around 50% of these lesions are < 5mm or microadenomas.

Pituitary apoplexy is uncommon and is secondary to sudden hemorrhage or infarction within a pituitary adenoma and it may result in visual loss or ophthalmoplegia. Computed tomography and MRI are essential in diagnosis as they will reveal hemorrhagic or necrotic parts of the pituitary tumor. Although pituitary apoplexy was considered a neurosurgical emergency, patients without important visual field defects and with preserved level of conscious can now be treated conservatively (13).

### MRI

**Figure 4:**
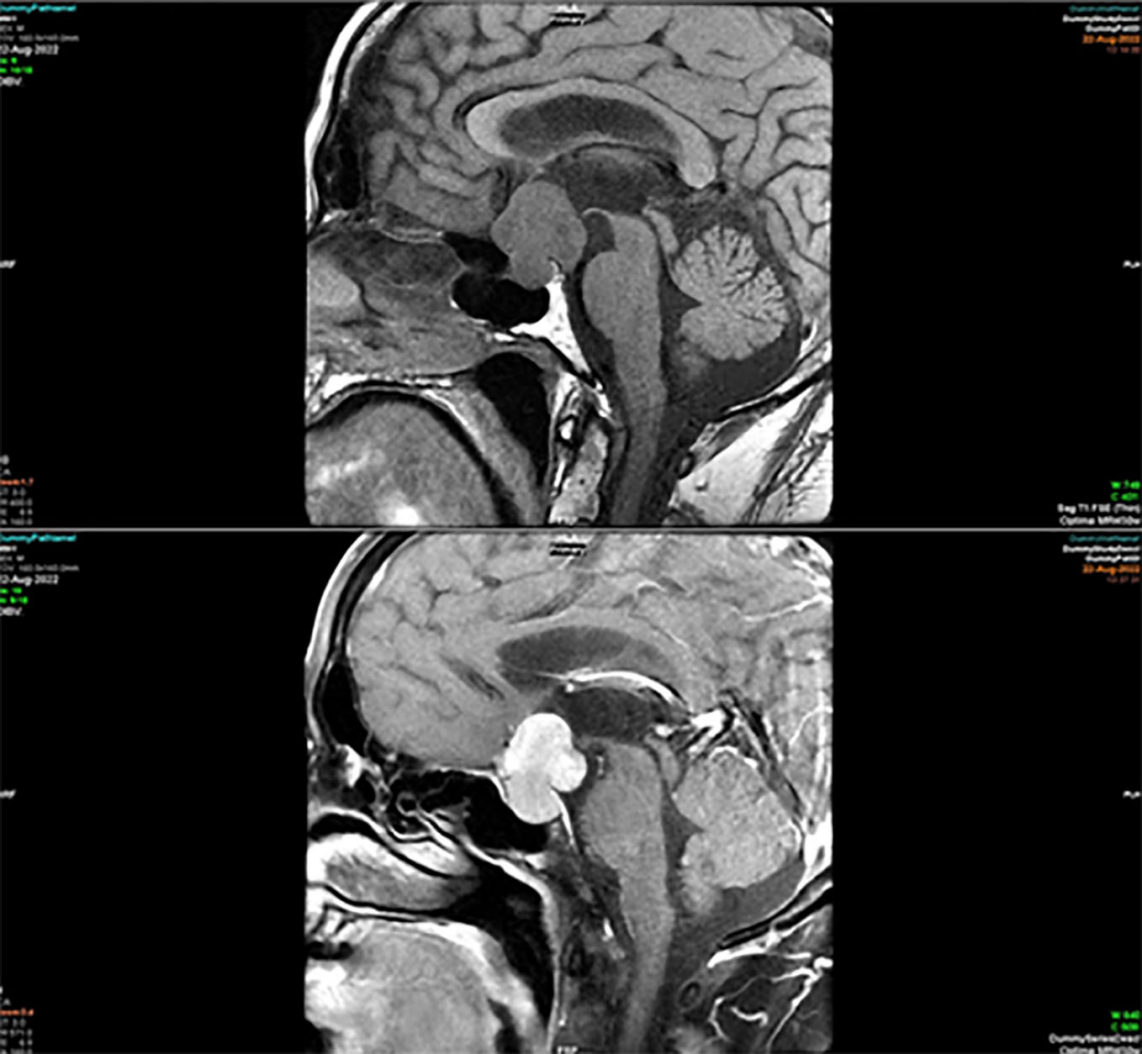
MRI T1 pre-contrast (top) and post-contrast (bottom) sagittal view of pituitary adenoma.

Typical features are isointense on T2 and T1 homogeneously except for large lesions that could be heterogeneous. Thin-section, contrast-enhanced MRI with dynamic contrast enhancement is considered the gold standard f or the diagnosis of the microadenoma (11, 14). Pituitary adenoma can become locally invasive, extending into the surrounding structures, i.e., hypothalamus, optic chiasm, ambient cistern, and temporal poles (**Figure 4**).

## Hormonal workup

Around 70% of pituitary adenomas are endocrine active, producing 1-2 hormones that may be detected in the blood and result in clinical symptoms. The rest are endocrine inactive (non-functional tumors), e.g., null cell adenoma, oncocytoma, and other less common types.

The most frequent hormone-secreting pituitary adenomas are prolactinomas (which account for 69% to 80% of all endocrine-secreting pituitary adenomas) (15). A diagnosis of prolactinoma is usually confirmed by serum prolactin levels >250 g/L combined with adenoma detection on high-resolution and postcontrast gradient echo (GRE) magnetic resonance (MR) imaging (16). Amenorrhea, galactorrhea, headache, infertility, mass effect on neighboring neurovascular systems, premature ejaculation, erectile dysfunction, and/or hypogonadism are some of the most common symptoms, which might vary depending on the gender of the patient. However, they can be frequently asymptomatic and diagnosed incidentally (17).

GH-secreting adenomas make up 13%–20% of all endocrine-secreting pituitary adenomas, and are more common in men. Acromegaly is usually confirmed by an increase in blood insulin-like growth factor-1 (IGF-1). An oral glucose tolerance test (OGTT) can be used in cases of equivocal IGF-1 values, and the absence of GH suppression to <1 μg/L is indicative of acromegaly (18). Before the closure of the epiphyseal growth plates, GH-secreting pituitary adenomas present with gigantism in around 5% of cases, and after, these tumors present with acromegaly. Features of acromegaly include excessive growth of hands and feet; enlargement of the nose and ears, mandibular prognathism, and coarsening of facial features. It also has additional systemic characteristics that negatively affect the patient’s quality of life.

ACTH-secreting pituitary adenomas (Cushing disease) make up 4.8% to 10% of all endocrine-secreting pituitary adenomas and are more common in women than in men (15). Weight gain (central obesity), diabetes, hypertension, moon facies, facial plethora, mental and neurocognitive abnormalities, diminished libido, and osteoporosis are all symptoms of Cushing’s disease. An adrenocortical tumor can be ruled out if ACTH levels are elevated. To distinguish between pituitary and ectopic sources of ACTH hypersecretion, high-dose dexamethasone suppression and corticotropin-releasing-hormone stimulation tests can be utilized.

Other less frequent endocrine active pituitary adenomas include TSH-secreting pituitary adenoma and gonadotroph pituitary adenomas (follicle-stimulating hormone [FSH] or luteinizing hormone [LH]-secreting).

## Craniopharyngioma

Craniopharyngiomas are benign slow-growing tumors with a point prevalence of about 2/100,000 (19). The onset is usually gradual, with symptoms including headaches and visual abnormalities, as well as endocrine (growth retardation and delayed puberty). Craniopharyngiomas are divided into two types: adamantinomatous, which is the most frequent in children, and papillary, which is the most common in adults. C raniopharngiomas are thought to be derived from epithelial remnants of the craniopharyngeal duct or Rathke’s pouch (adamantinomatous type) or metaplasia of squamous epithelial cell resting that contributed to the buccal mucosa (squamous papillary type). The differential diagnosis of tumors in this area include infectious or inflammatory processes (eosinophilic granuloma), vascular malformations (aneurysm), and congenital deformities (Rathke’s cleft cyst). The standard treatment is a gross complete excision of the tumor, or a subtotal resection with postoperative radiotherapy if there is hypothalamic invasion. The overall 5-year survival rate is 80%, albeit this is associated with significant morbidity (hypothalamic dysfunction, altered neuropsychological profile) (20).

### MRI

**Figure 5:**
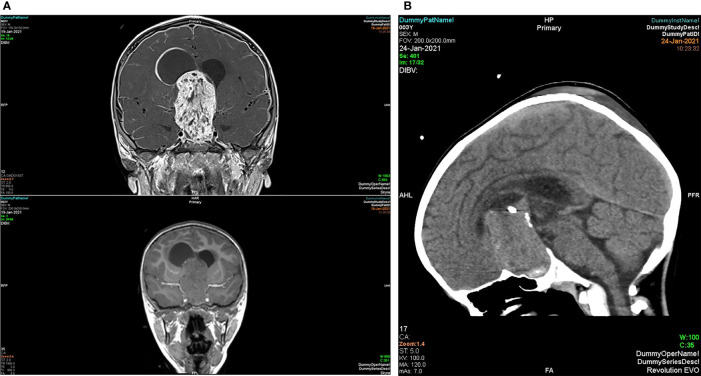
**(A)** MRI T1 with (top) and without (bottom) contrast coronal view of a giant craniopharyngioma with sellar and suprasellar solid enhancing parts; A large cystic component extends into the frontal horn of the right lateral ventricle. **(B)** A mid sagittal plane CT head of the same patient with evidence of sellar widening and peripheral calcification.

The size of the craniopharngiomas and, in particular, their extension to the hypothalamus, can be precisely determined by magnetic resonance imaging (MRI) with and without contrast. Depending on the cystic component, the MRI appearance may differ. The cystic components on T1-weighted sequences can be isointense to hyperintense to the brain hyperintensity, which is caused by the cyst fluid’s high protein content. The lesion shows a mixed hyperintense signal on T2-weighted sequences with enhancement the solid components of the tumor with contrast (see **Figure 5A**). MRA can be used to assess the tumor’s proximity to the internal carotid arteries and neighboring parts of the circle of Willis, which can be within the tumor. Magnetic resonance spectroscopy (MRS) is particularly useful in separating craniopharyngiomas from gliomas and pituitary adenomas; however, with smaller lesions around the skull base, this can be technically problematic. In addition. craniopharyngiomas show lactate or lipid peaks with only low levels of other metabolites on MRS, whereas gliomas show choline, n-acetyl aspartate, and creatine peaks, and pituitary adenomas show choline peaks or no increased metabolites (21).

On computed tomography (CT), calcification of the mass is best seen usually at the peripheral part of the tumors and the tumor might have a heterogeneous density with largely cystic and to lesser extent solid components (see **Figure 5B**). Calcification are typically seen in pediatric patients and in the histological adamantinomatous type, but not in the papillary type. Secondary changes to the skull base can be detected, such as the enlargement of the sella turcica or erosion of the dorsum sellae (22).

## Meningioma

Meningiomas are the most common intracranial extraaxial tumors, accounting for 13 to 26% of all primary intracranial tumors and just 1% of sellar masses. Additionally, 5-10% of all intracranial meningiomas are suprasellar/parasellar. They have gender predilection with a female- to-male ratio of 2.4:1 (23). The WHO’s grading system is divided into three categories. The majority of meningiomas are Grade I (low-grade), with Grade II and III meningiomas accounting for up to 20% of cases (high-grade). The most critical determinant in determining the clinical outcome, recurrence after resection and survival is the histopathological grade of the meningioma (24).

### MRI

**Figure 6:**
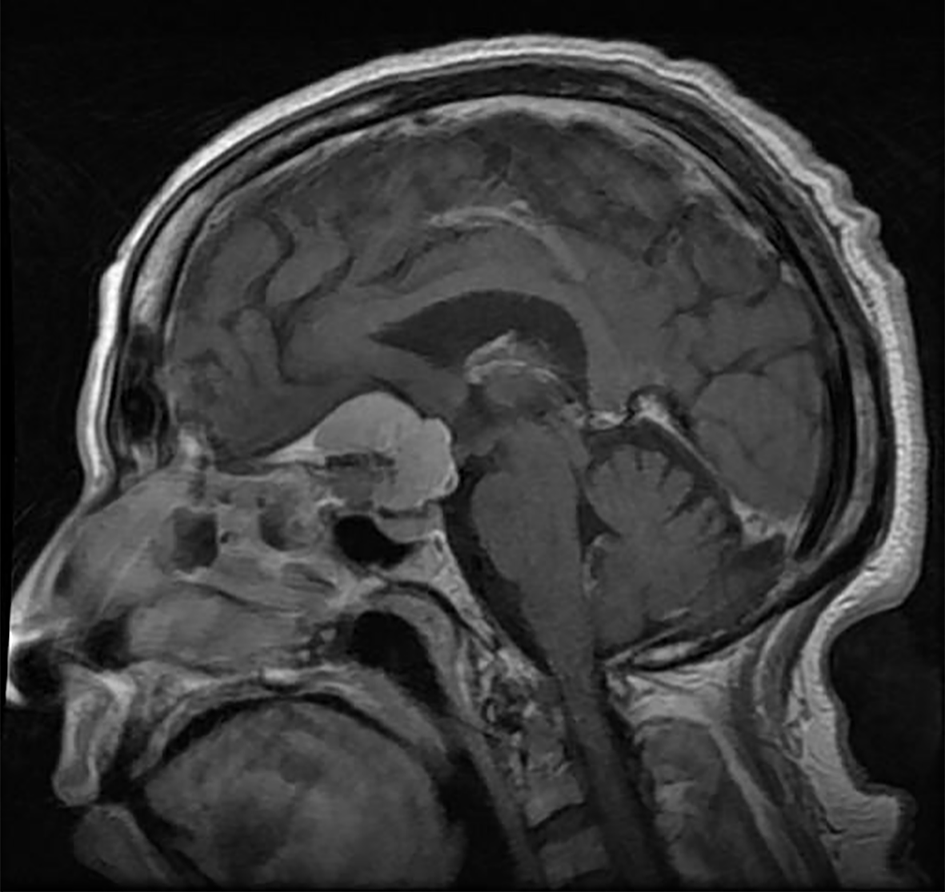
A midsagittal cut T1 with contrast demonstrating a sellar meningioma that has a clear dural tail anteriorly extending to the cribriform plate and normal adenophysis clearly demarcated intra sellarly.

Meningiomas are extra-axial masses that are lobular in shape and have well-defined borders with a broad-based dural attachment (**Figure 6**). They are usually isointense-hyperintense to cortical gray matter on T1-weighted images. However, on T2 they are homogenous to gray matter, unlike adenomas that are heterogeneous. The presence of calcification can give a hypointense appearance. After contrast administration, meningiomas typically demonstrate homogeneous bright enhancement on T1 with contrast. They frequently exhibit a “dural tail”, which is a linear enlargement of the neighboring dura. Heterogeneous enhancement, an unclear tumor-brain interface, and capsular enhancement can all be indicators of a high-grade meningioma (25) (**Figure 6**).

## Treatment

### Pituitary adenoma

#### Surgical treatment

The majority of pituitary tumors are still treated using the transsphenoidal surgical TSS method, which allows for pathologic evaluation and full or partial tumor excision (with the exception of prolactinomas, which should first receive medical treatment). The current standard technique for most non-functioning pituitary adenomas is endoscopy or microscopy-assisted transsphenoidal surgery. In some patients with a large pituitary tumor with invasion of nearby structures laterally, transcranial methods are still necessary. However, due to their complex anatomical position and diverse histology, pituitary tumors are difficult to surgically treat.

Compared to the conventional transsphenoidal microsurgical method, transsphenoidal endoscopic surgery (TSS) for functional pituitary adenomas yields better endocrinologic results for noninvasive macroadenomas. However, the rate of postoperative CSF leakage was greater with the endoscopic method (26).

In patients with pituitary adenomas, younger age, dense visual field defect, and the preoperative absence of central or bilateral visual field abnormalities were predictive factors of visual field recovery following transsphenoidal approach-tumor excision (27).

Although not generally accessible globally, intraoperative MRI reveals the tumor state during the procedure, allowing for the continuation of surgical removal of a tumor remnant.

Gross total resection can be achieved in 60–73% of individuals with non-functioning pituitary adenomas (NFPA). TSS was linked to 1% mortality in a recent meta-analysis of NFPA patients. Less than 5% of patients experienced postoperative complications like cerebrospinal fluid (CSF) leakage, fistula, meningitis, vascular injury, chronic diabetes insipidus, or new visual field defects. Patients with large adenomas with suprasellar extension, intraoperative CSF leakage, recurrent TSS, and high body mass index had a higher rate of CSF leakage (28). Hormone Replacement Therapy (HRT) is required often for those patients with endocrine disorder preoperatively or postoperatively when pituitary function is abnormal.

## Radiation treatment

Radiation therapy is recommended following subtotal resection of primary tumors such as macroadenomas, gross total excision of secreting adenomas with postoperative hormone secretion, and for recurrent tumors. Only patients with adenomas less than 25 to 30 mm and at least 2-3 mm from the chiasm are considered suitable for radiosurgery. With no evident differences between fractionated techniques and stereotactic radiosurgery, the choice of radiation technique should be based on size, location, and the availability of infrastructure and experience (SRS). Pituitary adenoma radiation (EBRT) dose recommendations range from 45 to 50.4 Gy, administered over 25 to 28 fractions over the course of 5 to 6 weeks utilizing current, high-precision methods. For secretory tumors and non-functioning adenomas the recommended doses of stereotactic radiosurgery are 16–20 Gy and 12–14 Gy, respectively (29).

With the introduction of highly conformal stereotactic methods as well as innovative planning and dose delivery strategies, such as intensity-modulated radiotherapy (IMRT) and volumetric modulated arc therapy (VMAT), radiation therapy has advanced. All of these modern approaches enable accurate, targeted radiation administration, lowering the dose to nearby vital neurovascular and cerebral structures and possibly minimizing the long-term effects of radiation therapy (30). For example, Hypopituitarism, neurocognitive impairment, neuropsychological dysfunction, optic neuropathy, radiation necrosis of the chiasm, radiation retinopathy, cerebrovascular accidents, and secondary malignant neoplasms are among the delayed side-effects of pituitary radiotherapy.

External beam radiation therapy (EBRT) has been shown to be a viable treatment option for pituitary adenoma which are resistant to surgery and/or medical therapy, with excellent long-term local control (> 90%) in 10 years, but lower rates (50–80%) of biochemical remission in secretory tumors (31). Another therapeutic option is proton therapy, which specifically targets the tumor while the normal tissue is protected. As a result, it may decrease long-term side effects such as secondary malignancies.

## Craniopharyngioma

### Timing of therapy

This mainly concerns pediatric cases with the delay of definitive treatment in prepubescent cases due to narrow nasal passage, that could affect ideal endonasal resection. It is important to consider the timing of therapy and to delay exposure of the prepubescent brain to radiation that can affect the hypothalmic-pituitary-axis (HPA) and thus allow the patient to enter puberty. In the case of largely cystic tumors, an insertion of an intracystic catheter to decompress the cyst and relieve potential pressure on the optic and CSF pathways can be done, with beneficial effects lasting months or years. These catheters can also enable injection of therapeutic agents Interferon-alpha (IFNa) and other radionuclide materials, e.g. 90Y and 32 P, that have had favorable results in control of cystic growth.

## Surgical treatment

With the goal of preserving hypothalamic-pituitary and optic nerve functions, total resection is the therapy of choice for patients with good tumor location. A limited resection followed by local irradiation is advised when the tumor localization is unfavorable, that is, when the tumor causes the hypothalamus to become dislocated, compressed, or invaded starting at the level of the mammillary bodies or dorsal to them. Survival rates are high generally (91-98%) (32).

Endoscopic endonasal surgery is no longer reserved primarily for sellar or minor cystic suprasellar lesions This method can be used to eliminate prechiasmatic/preinfundibular and subchiasmatic/transinfundibular ones. Endoscopic endonasal surgery combines the benefits of the caudocranial and midline approaches, allowing for appropriate infra chiasmatic exposure without the need to manipulate nearby neurovascular structures to access the tumor, in contrast to well-established transcranial approaches (pterional, subfrontal, and presigmoid). This anatomic advantage, along with high-definition wide-angle imaging, excellent endonasal microsurgical procedures, and instrumentation permits a high rate of endocrine function maintenance and visual improvement, while concurrently obtaining comparable resections. Endoscopic skull base restoration with the vascularized nasoseptal flap has drastically reduced the rate of cerebrospinal fluid leak, reinforcing endoscopic endonasal surgery as a successful and safe alternative for the treatment of these challenging lesions (33). The endoscopic endonasal method has benefits for the treatment of craniopharyngiomas that have been managed using the transsphenoidal route (i.e., purely intrasellar or intra-suprasellar infradiaphragmatic, preferably cystic lesions in patients with panhypopituitarism). The use of the “extended” endoscopic endonasal technique overcomes the limitations of the transsphenoidal route to the sella, enabling the management of various purely suprasellar and retrosellar cystic/solid craniopharyngiomas regardless of the sellar size or pituitary function (34).

## Radiation treatment

After partial resection surgery, patients who receive radiotherapy (RT) have great long-term tumor control. High recurrence rates, especially after incomplete resection, have been observed, thus requiring close post-operative follow-up or immediate radiosurgery. As a more precise irradiation approach with more accurate tumor localization, stereotactic radiotherapy—both in the form of radiosurgery (RS) and fractionated stereotactic radiotherapy (FSRT)—has been developed. All sizes of craniopharyngiomas can be treated with FSRT, and its effectiveness is on equal to traditional RT. Single-fraction stereotactic radiosurgery is commonly given to small tumors away from important structures. To validate the excellent tumor control and the possible reduction in long-term radiation harm, a lengthier follow-up is required (35). Proton radiotherapy, another technique gaining popularity theoretically, spares surrounding normal tissue exposing it to significantly less low volume radiation compared to photon methods. A more recent type of proton therapy (pencil-beam scanning) has been made accessible since 2016. The use of small, individually weighted beams, which further adapt the prescribed dose to the target and minimize both the volume that receives the highest doses and the volume that receives the lowest doses, gives this second-generation approach an advantage over the first generation. Long term follow up clinical trials are still pending.

## Alternative treatment

Research is ongoing on administration of BRAF v600E specific inhibitors, such as Dabrafenib and Vemurafenib, with the addition of MEK inhibitors, including Trametinib and Cobimetinib, for cases of papillary craniopharyngioma.

## Meningioma

Meningiomas that do not display a mass effect or are asymptomatic can be observed. Patients with known grade 1 meningiomas had mean absolute growth rates of 1.51 cm (3)/year, median relative growth rates of 14.18 percent%/year, and tumor doubling times of 5.228 years, respectively, with a stronger trend in younger patients (36). The combination of surgery and radiosurgery or fractionated RT is being more used.

## Surgical treatment

A gross total surgical resection which includes the involved dura is theraputic. This can be difficult in skull base tumors such as the sellar ones. The potential for neurological damage to patients must be weighed against the decision to completely remove meningiomas and affected tissues. The approach being the standard transcranial, e.g., pterional, subfrontal. For suprasellar meningiomas, trans-nasal endoscopic surgery is a successful method that, in the right circumstances, yields tumor removal outcomes that are comparable to those of the transcranial approach. Visual results were superior, and vascularized flaps significantly decreased rates of CSF leak (37)

In the group who underwent surgical treatment for an intracranial meningioma, the probability of needing a second operation due to a recurrence was 1% per patient-year of follow-up. One of the most important predictors of the rate of recurrence has been shown to be the extent of resection, i.e. Simpson grade of resection. A subgroup of meningiomas, most frequently those in grades II and III, exhibit aggressive behavior and early recurrences that are challenging to cure despite considerable surgical resection.

## Radiation treatment

If the patient’s general medical condition obviates surgery or if the procedure carries high risk of damage to adjacent vital structures, then stereotactic radiosurgery or fractionated radiotherapy can be performed. Recurrent tumors can be managed with radiosurgery or fractionated radiotherapy depending on their size and proximity to important structures since re-operation is known to be associated with higher morbidity and mortality. Post-operative management and treatment plan is dictated by the histology grade and degree of resection. WHO grade 1, gross total resection should be followed by observation, especially Simpson grade1 resection, i.e. complete resection. WHO grade 1, subtotal or partial resection can be observed for recurrence or followed immediately with stereotactic radiosurgery or fractionated radiotherapy to decrease the chance of recurrence in a location which could be difficult to treat, eg. sellar or skull base location. WHO grade II, gross total resection could be followed by observation or fractionated radiotherapy. Adjuvant radiotherapy had been found to delay recurrence and improved overall survival (OS). Lastly, WHO grade III will require adjuvant treatment consisting of fractionated radiotherapy and potential chemotherapy, clinical trials or immunotherapy due to its high rate of recurrence with surgery alone. Somatostatin receptor (SSTR)–targeted peptide receptor radionuclide therapy (PRRT) has been shown to have around 63% disease control in those meningiomas resistant to standard treatment (38).

## Conclusion

Sellar tumors are diagnosed by neuro-ophthalmic examinations, investigations, and neuro-imaging, mainly MRI. OCT can be used not only in the diagnosis of pituitary tumors, but also a prognostic predictor following surgery or even hormonal treatment in the case of prolactinomas. Loss of ganglion cell layer complex can be seen with chiasmal compression, and patients with less GCC loss before decompression had better outcome in visual field recovery. Transsphenoidal surgery is the mainstay of treatment of most pituitary tumors, and endoscopic transsphenoidal surgery leads to better endocrinologic outcomes than traditional microsurgical techniques. There are different types of radiotherapy, external radiation therapy and stereotactic radiosurgery, and the decision should be made based on the features of the tumor. Radiosurgery is especially useful if the tumor is recurrent or inoperable. Treatment of sellar tumors usually combines surgery and radiosurgery to achieve the best outcome.

## Author contributions

DA-B contributed as first author in the study concept and data acquisition, as well as data analysis and interpretation. RB was responsible for data on neuro-ophthalmology and review as well as quality assessment. AH was responsible for review of neurosurgery data and editing. All authors were responsible in drafting the manuscript. All authors contributed to the article and approved the submitted version. 

## Acknowledgments

Dr. Thomash Hedges (New England Eye Center, Boston, MA) and Dr. Eric Berman (Storm Eye Institute, South Carolina) for providing some of the OCT images and visual fields Dr. Lamya AlSarraf and Dr. Fatima Dashti for providing clinical images to support this article.

## Conflict of interest

The authors declare that the research was conducted in the absence of any commercial or financial relationships that could be construed as a potential conflict of interest.

## Publisher’s note

All claims expressed in this article are solely those of the authors and do not necessarily represent those of their affiliated organizations, or those of the publisher, the editors and the reviewers. Any product that may be evaluated in this article, or claim that may be made by its manufacturer, is not guaranteed or endorsed by the publisher.

## References

[1] Subhawong TK Fishman EK Swart JE Carrino JA Attar S Fayad LM . Soft-tissue masses and masslike conditions: what does CT add to diagnosis and management? AJR Am J Roentgenol (2010) 194(6):1559–67. doi: 10.2214/AJR.09.3736 PMC2884142 20489097

[2] Ogra S Nichols AD Stylli S Kaye AH Savino PJ Danesh-Meyer HV . Visual acuity and pattern of visual field loss at presentation in pituitary adenoma. J Clin Neurosci (2014) 21(5):735–40. doi: 10.1016/j.jocn.2014.01.005 24656736

[3] Rebolleda G Diez-Alvarez L Casado A Sánchez-Sánchez C de Dompablo E González-López JJ . OCT: New perspectives in neuro-ophthalmology. Saudi J Ophthalmol (2015) 29(1):9–25. doi: 10.1016/j.sjopt.2014.09.016 25859135 PMC4314576

[4] Vuong LN Hedges TR 3rd . Ganglion cell layer complex measurements in compressive optic neuropathy. Curr Opin Ophthalmol (2017) 28(6):573–8. doi: 10.1097/ICU.0000000000000428 28984725

[5] Tieger MG Hedges TR 3rd Ho J Erlich-Malona NK Vuong LN Athappilly GK . Ganglion cell complex loss in chiasmal compression by brain tumors. J Neuroophthalmol (2017) 37(1):7–12. doi: 10.1097/WNO.0000000000000424 28192385 PMC6033516

[6] Danesh-Meyer HV Carroll SC Foroozan R Savino PJ Fan J Jiang Y . Relationship between retinal nerve fiber layer and visual field sensitivity as measured by optical coherence tomography in chiasmal compression. Invest Ophthalmol Vis Sci (2006) 47(11):4827–35. doi: 10.1167/iovs.06-0327 17065494

[7] Glebauskiene B Liutkeviciene R Zlatkute E Kriauciuniene L Zaliuniene D . Association of retinal nerve fibre layer thickness with quantitative magnetic resonance imaging data of the optic chiasm in pituitary adenoma patients. J Clin Neurosci (2018) 50:1–6. doi: 10.1016/j.jocn.2018.01.005 29398198

[8] Wang MTM King J Symons RCA Stylli SS Meyer J Daniell MD . Prognostic utility of optical coherence tomography for long-term visual recovery following pituitary tumor surgery. Am J Ophthalmol (2020) 218:247–54. doi: 10.1016/j.ajo.2020.06.004 32533947

[9] Meyer J Diouf I King J Drummond K Stylli S Kaye A . Symons RCA. a comparison of macular ganglion cell and retinal nerve fibre layer optical coherence tomographic parameters as predictors of visual outcomes of surgery for pituitary tumours. Pituitary (2022) 25(4):563–72. doi: 10.1007/s11102-022-01228-w 35552990

[10] Yoo YJ Hwang JM Yang HK Joo JD Kim YH Kim CY . Prognostic value of macular ganglion cell layer thickness for visual outcome in parasellar tumors. J Neurol Sci (2020) 414:116823. doi: 10.1016/j.jns.2020.116823 32302803

[11] Smith KJ Blakemore WF McDonald WI . Central remyelination restores secure conduction. Nature (1979) 280(5721):395–6. doi: 10.1038/280395a0 460414

[12] Daly AF Beckers A . The epidemiology of pituitary adenomas. Endocrinol Metab Clin North Am (2020) 49(3):347–55. doi: 10.1016/j.ecl.2020.04.002 32741475

[13] Briet C Salenave S Chanson P . Pituitary apoplexy. Endocrinol Metab Clin North Am (2015) 44(1):199–209. doi: 10.1016/j.ecl.2014.10.016 25732655

[14] Rand T Lippitz P Kink E Huber H Schneider B Imhof H . Evaluation of pituitary microadenomas with dynamic MR imaging. Eur J Radiol (2002) 41(2):131–5. doi: 10.1016/s0720-048x(01)00412-0 11809542

[15] Tjörnstrand A Gunnarsson K Evert M Holmberg E Ragnarsson O Rosén T . The incidence rate of pituitary adenomas in western Sweden for the period 2001-2011. Eur J Endocrinol (2014) 171(4):519–26. doi: 10.1530/EJE-14-0144 25084775

[16] Melmed S Casanueva FF Hoffman AR Kleinberg DL Montori VM Schlechte JA . Diagnosis and treatment of hyperprolactinemia: an endocrine society clinical practice guideline. J Clin Endocrinol Metab (2011) 96(2):273–88. doi: 10.1210/jc.2010-1692 21296991

[17] Carter JN Tyson JE Tolis G Van Vliet S Faiman C Friesen HG . Prolactin-secreting tumors and hypogonadism in 22 men. N Engl J Med (1978) 299(16):847–52. doi: 10.1056/NEJM197810192991602 211411

[18] Katznelson L Laws ER Jr Melmed S Molitch ME Murad MH Utz A . Acromegaly: an endocrine society clinical practice guideline. J Clin Endocrinol Metab (2014) 99(11):3933–51. doi: 10.1210/jc.2014-2700 25356808

[19] Bunin GR Surawicz TS Witman PA Preston-Martin S Davis F Bruner JM . The descriptive epidemiology of craniopharyngioma. J Neurosurg (1998) 89(4):547–51. doi: 10.3171/jns.1998.89.4.0547 9761047

[20] Garnett MR Puget S Grill J Sainte-Rose C . Craniopharyngioma. Orphanet J Rare Dis (2007) 2:18. doi: 10.1186/1750-1172-2-18 17425791 PMC1855047

[21] Verma A Kumar I Verma N Aggarwal P Ojha R . Magnetic resonance spectroscopy - revisiting the biochemical and molecular milieu of brain tumors. BBA Clin (2016) 5:170–8. doi: 10.1016/j.bbacli.2016.04.002 PMC4845155 27158592

[22] Kucharczyk W Truwit CL . Diseases of the sella turcica and parasellar region. In: Hodler J Kubik-Huch RA von Schulthess GK , editors. Diseases of the brain, head and neck, spine 2020–2023: Diagnostic imaging. Cham (CH: Springer (2020). 32119253

[23] Chaudhry SK Raza R Naveed MA Rehman I . Suprasellar meningiomas: An experience of four cases with brief review of literature. Cureus (2021) 13(1):e12470. doi: 10.7759/cureus.12470 33552786 PMC7854335

[24] Harter PN Braun Y Plate KH . Classification of meningiomas-advances and controversies. Chin Clin Oncol (2017) 6(Suppl 1):S2. doi: 10.21037/cco.2017.05.02 28595423

[25] Watts J Box G Galvin A Brotchie P Trost N Sutherland T . Magnetic resonance imaging of meningiomas: a pictorial review. Insights Imaging (2014) 5(1):113–22. doi: 10.1007/s13244-013-0302-4 PMC3948902 24399610

[26] D'Haens J Van Rompaey K Stadnik T Haentjens P Poppe K Velkeniers B . Fully endoscopic transsphenoidal surgery for functioning pituitary adenomas: a retrospective comparison with traditional transsphenoidal microsurgery in the same institution. Surg Neurol (2009) 72(4):336–40. doi: 10.1016/j.surneu.2009.04.012 19604551

[27] Lee DK Sung MS Park SW . Factors influencing visual field recovery after transsphenoidal resection of a pituitary adenoma. Korean J Ophthalmol (2018) 32(6):488–96. doi: 10.3341/kjo.2017.0094 PMC6288018 30549473

[28] Esposito D Olsson DS Ragnarsson O Buchfelder M Skoglund T Johannsson G . Non-functioning pituitary adenomas: indications for pituitary surgery and post-surgical management. Pituitary (2019) 22(4):422–34. doi: 10.1007/s11102-019-00960-0ssd PMC6647426 31011999

[29] Becker G Kocher M Kortmann RD Paulsen F Jeremic B Müller RP . Radiation therapy in the multimodal treatment approach of pituitary adenoma. Strahlenther Onkol (2002) 178(4):173–86. doi: 10.1007/s00066-002-0826-x 12040754

[30] Teoh M Clark CH Wood K Whitaker S Nisbet A . Volumetric modulated arc therapy: a review of current literature and clinical use in practice. Br J Radiol (2011) 84(1007):967–96. doi: 10.1259/bjr/22373346 PMC3473700 22011829

[31] Gupta T Chatterjee A . Modern radiation therapy for pituitary adenoma: Review of techniques and outcomes. Neurol India (2020) 68(Supplement):S113–22. doi: 10.4103/0028-3886.287678 32611901

[32] Müller HL . Childhood craniopharyngioma–current concepts in diagnosis, therapy and follow-up. Nat Rev Endocrinol (2010) 6(11):609–18. doi: 10.1038/nrendo.2010.168 20877295

[33] Fernandez-Miranda JC Gardner PA Snyderman CH Devaney KO Strojan P Suárez C . Craniopharyngioma: a pathologic, clinical, and surgical review. Head Neck (2012) 34(7):1036–44. doi: 10.1002/hed.21771 21584897

[34] Cavallo LM Solari D Esposito F Villa A Minniti G Cappabianca P . The role of the endoscopic endonasal route in the management of craniopharyngiomas. World Neurosurg (2014) 82(6 Suppl):S32–40. doi: 10.1016/j.wneu.2014.07.023 25496633

[35] Minniti G Esposito V Amichetti M Enrici RM . The role of fractionated radiotherapy and radiosurgery in the management of patients with craniopharyngioma. Neurosurg Rev (2009) 32(2):125–32. doi: 10.1007/s10143-009-0186-4 19165514

[36] Nakamura M Roser F Michel J Jacobs C Samii M . Volumetric analysis of the growth rate of incompletely resected intracranial meningiomas. Zentralbl Neurochir. (2005) 66(1):17–23. doi: 10.1055/s-2004-836225 15744624

[37] Gupta PP Shaikh ST Deopujari CE Shah NJ . Transnasal endoscopic surgery for suprasellar meningiomas. Neurol India (2021) 69(3):630–5. doi: 10.4103/0028-3886.319224 34169857

[38] Goldbrunner R Stavrinou P Jenkinson MD Sahm F Mawrin C Weber DC . EANO guideline on the diagnosis and management of meningiomas. Neuro Oncol (2021) 23(11):1821–34. doi: 10.1093/neuonc/noab150 PMC8563316 34181733

